# Molecular identification and subtype analysis of *Blastocystis* in captive Asiatic black bears (*Ursus thibetanus*) in China’s Heilongjiang and Fujian provinces

**DOI:** 10.3389/fcimb.2022.993312

**Published:** 2022-08-29

**Authors:** Yuting-Li Zhou, Nairui Zhao, Yilei Yang, Yueqi Li, Xinyu Zhang, Jiani Chen, Xiang Peng, Wei Zhao

**Affiliations:** ^1^ School of Basic Medicine, Wenzhou Medical University, Wenzhou, China; ^2^ Second School of Clinical Medicine of the Wenzhou Medical University, Wenzhou, China

**Keywords:** genotypic variation, zoonotic, bear, *Blastocystis*, subtype

## Abstract

*Blastocystis* sp. is the most isolated enteric protozoan in parasitological surveys of humans. A substantial percentage of human infections is attributed to zoonotic transmissions. However, the contribution of each animal source to human infections with blastocystis is not yet fully understood. This study thus aimed to determine the infection rates and subtype distributions of *Blastocystis* sp. in captive Asiatic black bears (*Ursus thibetanus*) in China’s Heilongjiang and Fujian provinces. A total of 218 fresh fecal specimens were collected from captive Asiatic black bears in Heilongjiang (n = 36) and Fujian (n = 182) between May 2015 and December 2017. Genomic DNA was extracted from each sample and then examined for *Blastocystis* through SSU rRNA gene amplicon-based sequencing. A phylogenetic tree based on the *Blastocystis* positive sequences was reconstructed using the Mega X program. Eleven percent (24/218) of the animals had *Blastocystis* and six *Blastocystis* subtypes, including ST4 (n = 14), ST10 (n = 3), ST1 (n = 2), ST2 (n = 1), ST5 (n = 1), and ST12 (n = 1) were identified. A total of 14 representative sequences, including seven sequences that have been described previously and seven novel sequences comprising ST10 (n = 2), ST5 (n = 1), and ST4 (n = 4), were obtained from the six subtypes of *Blastocystis*. This study is the first to report the presence of *Blastocystis* in captive Asiatic black bears in Fujian, China. It provides baseline data for controlling and preventing *Blastocystis* infection in farm communities. Zoonotic infections in bears with ST1, ST2, ST4, ST5, ST10, and ST14 should be considered potential public health threats. The novel ST sequences of *Blastocystis* generated in this study provide novel insights into the genotypic variation within the *Blastocystis* sp.

## Introduction

*Blastocystis* sp. is a common zoonotic pathogen that inhabits the human and animal intestines and has been reported worldwide (Rauff-Adedotun et al., 2021). Previously, it was classified as yeast, fungus, or cyst stage of another organism. However, it was classified as a protozoon belonging to the genus *Blastocystis* of the Stramenopiles group 25 years ago (Silberman et al., 1996). Recent years have seen much controversy about the pathogenicity of *Blastocystis*. Although many studies have determined *Blastocystis* infection is generally asymptomatic, patients infection with this organism appear symptoms characterized by diarrhea, abdominal pain, weight loss, and excess gas in other investigations (Rezaei Riabi et al., 2017; Robles-Cabrera et al., 2021). Moreover, infection with *Blastocystis* can lead to severe symptoms and even death in immunocompromised patients (Khorshidvand et al., 2021). It is generally believed that the most probable infections of *Blastocystis* sp. result from fecal-oral transmission of oocysts from infected humans or animals through contaminated food and water (Rauff-Adedotun et al., 2021). It is postulated that a percentage of human infections are a consequence of zoonotic transmission because infections are common in certain occupations that involve exposure to animals, including food and animal handlers, such as zookeepers and abattoir workers (Tan, 2008). One of the key questions in controlling the infection of *Blastocystis* is to affirm the source of infection and transmission dynamics. Infection with *Blastocystis* is universally recognized as a global public health concern because *Blastocystis* spp. are common in a range of animals with close contact with humans (Hublin et al., 2021; Popruk et al., 2021). However, the contribution of each animal source to human infections remains unclear, thus requiring elucidation.

Previously, *Blastocystis* infections were primarily diagnosed through light microscopic examination of fecal material. Currently, PCR-based methods are widely used to detect and characterize *Blastocystis* to the molecular level (Stensvold and Clark, 2016a). These methods offer higher specificity and sensitivity and are advantageous in identifying genotypes. Nowadays, genotyping of *Blastocystis* mainly relies on analyzing the polymorphic regions of its small subunit of the ribosomal RNA (SSU rDNA) gene (Stensvold and Clark, 2016b). To date, *Blastocystis* has been classified into 28 subtypes designated as ST1-ST17, ST21, ST23-ST29, and ST30-ST32 based on the SSU rDNA gene sequences (Wang et al., 2022). Among them, ST1–ST10, ST12, ST14, ST16, and ST26 have been reported in both humans and animals, indicating their zoonotic potential (Hublin et al., 2021; Popruk et al., 2021). The remaining STs have been reported only in animals, suggesting that they are host specific and have a reduced risk to public health (Hublin et al., 2021; Popruk et al., 2021). Subtyping of *Blastocystis* spp is important to identify potential sources and routes of transmission. Epidemiological surveys should thus focus on genotyping *Blastocystis* isolates from the animal hosts that frequently infect humans to improve our understanding of the epidemiology of human blastocystiosis and strengthen our knowledge of *Blastocystis* animal populations during human transmission.

In China, *Blastocystis* has been reported in humans distributed in at least 12 provinces. Moreover, twenty-five animal species from eight provinces have also been reported to be infected with *Blastocystis* (Deng et al., 2019; Ning et al., 2020). These studies are mainly drawn from the west and northeast of China, with the *Blastocystis* STs in other provinces in China remaining not fully understood. Asiatic black bears are widely kept on farms for their economic and medical value and are also commonly found in zoos as commercial and ornamental animals in China. They are in close contact with humans, including farmers, animal keepers, vets, visitors, and zookeepers. Exposure to infective cysts of *Blastocystis* from the feces of Asiatic black bears poses a potential risk to other animals and public health because of the high-density feeding environment in farms and zoos. Currently, there are only two reports describing *Blastocystis* infection in bears from Heilongjiang and Sichuan Provinces in China (Ni et al., 2021; Deng et al., 2021). This study thus aimed to determine the infection rates and subtype distributions of *Blastocystis* in confined Asiatic black bears through PCR amplification and analysis of the Santín regions of the SSU rRNA gene. The study’s ultimate focus was to understand the genetic characterizations of the *Blastocystis* isolates and assess their zoonotic potential.

## Materials and methods

### Ethics statement

The protocol of this study was reviewed and approved by the Research Ethics Committee and the Animal Ethical Committee of Wenzhou Medical University. All fecal samples were obtained from feces defecated by animals with the approval of the animals’ owners or managers. No animals were hurt during the procedure.

### Collection of fecal specimens

Between May 2015 and December 2017, approximately 50 g of fresh feces were collected from 218 Asiatic black bears in a zoo in the Heilongjiang (n = 36) and a farm in the Fujian (n = 182) Provinces of China ​(**Table 1**). The sampling farms were selected based on the owners’ willingness to participate and the accessibility of animals for sampling. The number of collected specimens accounted for approximately 30% of bears on the farm. The fecal specimens were collected from the ground directly after defecation. The specimens were collected using sterile disposable latex gloves and were placed in a labelled sterile bag. The specimens were then transported to the laboratory and stored at 4°C until processing, which was done within 12 h.

**Table 1:** Prevalence and ST subtypes of *Blastocystis* in bears from two provinces according to age, gender and feeding mode.

Group	Fujian		Heilongjiang	
	Positive no./Examined no. (%)	ST subtypes (n)	Positive no./Examined no. (%)	ST subtypes (n)
Age (year)				
<3	4/17 (23.5)	ST4 (4)	4/12 (33.3)	ST10 (3), ST14 (1)
3-5	13/122 (10.7)	ST4 (11); ST2 (1), ST5 (1)	–	–
>5	1/43 (2.3)	ST1 (1)	2/24 (8.3)	ST1 (1); ST10 (1)
Gender				
Male	13/118 (11.0)	ST4 (12); ST2 (1)	3/15 (20.0)	ST10 (3)
Female	5/64 (7.8)	ST4 (3); ST1 (1), ST5 (1)	3/21 (14.3)	ST1 (1); ST10 (1), ST14 (1)
Feeding mode				
Alone	6/92 (6.5)	ST4 (3); ST1 (1), ST2 (1); ST5 (1)	6/36 (16.7)	ST1 (1); ST10 (4), ST14 (1)
Group	12/90 (13.3)	ST4 (12)	–	–
Total	18/182 (9.9)	ST4 (15); ST1 (1), ST2 (1); ST5 (1)	6/36 (16.7)	ST1 (1); ST10 (4), ST14 (1)

### Information records

Information on the Asiatic black bears, including age, sex, and with or without diarrhea, were acquired from the trainers and tabulated in MS Excel 2007 database templates. Among the 182 bears from Fujian Province, 17, 122, and 43 were aged < 3 years, 3-5 years, and >5 years, respectively. In addition, there were 118 male bears and 64 female bears, amongst which 92 bears were raised individually and 90 were raised in a group. In Heilongjiang Province, 12 bears were aged < 3 years while the remaining 24 were aged > 5 years. All the 36 bears were raised individually and comprised 15 males and 21 females. None of the animals showed diarrhea at the time of sample collection.

### DNA extraction

The fecal specimens were sieved through an 8.0-cm-diameter sieve with a pore size of 45 μm, followed by concentrating the sift through centrifugation at 1,500 g for 10 min. Genomic DNA was then directly extracted from 200 mg of the sift fecal specimens using a QIAamp DNA stool mini kit (Qiagen, Hilden, Germany) following the manufacturer’s -recommended procedures. The extracted DNA was stored at −20°C and was subsequently used for PCR analysis.

### PCR amplification

The DNA samples were analyzed for the presence of *Blastocystis* by amplifying the Santín region, a 500 bp nucleotide fragment, of the SSU rRNA gene of *Blastocystis*. The primers used and the cycling conditions were used as described by Santín et al. (2011). TaKaRa Taq DNA Polymerase (TaKaRa Bio Inc., Tokyo, Japan) was used for all PCR amplifications. A negative control was included in all PCR tests to check for low-level contamination. The PCR products were subjected to 1.5% agarose gel electrophoresis and were visualized on a Gel Doc EZ UV-gel imaging system (Bio-Rad Inc., USA). The gel was stained with GelRed (Biotium Inc., Hayward, CA) to aid visualization.

### Nucleotide sequencing and analysis

We used the Big Dye Terminator v3.1 Cycle Sequencing Kit (Applied Biosystems, USA) to sequence the positive nested PCR products after being purified on an ABI PRISM 3730 XL DNA Analyzer (Sino Geno Max Biotechnology Co., Ltd., Beijing, China). Sequence accuracy was confirmed by two-directional sequencing and sequencing of additional PCR products for some DNA samples where necessary. We subsequently used DNASTAR Lasergene EditSeq v7.1.0 (http://www.dnastar.com/) to edit the derived sequences and Clustal X v2.1 (http://www.clustal.org/) to align them with reference sequences downloaded from the GenBank.

### Phylogenetic analysis

A phylogenetic tree based on the neighboring-joining method and Kimura-2-parameter model was reconstructed using the Mega X (http://www.megasoftware.net) software to assess the genetic relationship between the subtypes of *Blastocystis*. The reliability of the tree was assessed through bootstrap analysis with 1,000 replicates.

### Statistical analyses

Statistical analyses were performed using SPSS version 22.0 (SPSS Inc., Chicago, IL, USA). The Chi-square test or Fisher’s Exact test at a 95% confidence interval were used, where applicable, to compare the prevalence of *Blastocystis* among different ages, gender, and feeding mode groups within each province. Differences between groups were considered statistically significant when *P*-values were < 0.05.

## Results

### Infection rates of *Blastocystis*

PCR amplification of the Santín region of the SSU rRNA gene revealed that 24 (11.0%) of the 218 fecal specimens were positive for *Blastocystis*. Specifically, 18 of 182 (9.9%) and 6 of 36 (16.7%) bears sampled from the farm in Fujian and the zoo in Heilongjiang, respectively, were *Blastocystis* sp. positive (**Table 1**). The difference in *Blastocystis* sp. prevalence between the farm and zoo was insignificant (χ^2^ = 0.80, df = 1, *P* = 0.37). Among the three age groups in the Fujian farm, the prevalence of *Blastocystis* in the <3 years group was 23.5%, which was higher than those in the 3-5 years (10.7%) and >5 years (2.3%) groups. Notably, the difference was statistically significant (*P* = 0.04). In the same line, the prevalence of *Blastocystis* in males on the farm was 11.0%, which was higher than that in females (7.8%), but not significantly different (χ^2^ = 0.49, df = 1, *P* = 0.49). Between the two feeding mode groups, the prevalence of *Blastocystis* in the group feeding was 13.3%, double that of individual feeding (6.5%). Similar to the age groups in Fujian farm, the prevalence of *Blastocystis* in the <3 years group in Heilongjiang zoo was 33.3%, which was higher than that in the >5 years (8.3%) (*P* = 0.15). The prevalence of *Blastocystis* in males in the zoo was 20.0%, which was higher than that in females (14.3%), but not significantly different (*P* = 0.68).

### Subtypes of *Blastocystis* isolates

Six *Blastocystis* subtypes were identified out of 24 *Blastocystis* isolates after sequence analysis of the Santín region. The subtypes included ST4 (n = 15), ST10 (4), ST1 (n = 2), ST2 (n = 1), ST5 (n = 1), and ST14 (n = 1). ST1 was detected in animals from both Heilongjiang and Fujian provinces, ST14, ST2, and ST5 were only detected in animals from Fujian, and ST10 and ST14 were only detected in animals from Heilongjiang (**Table 1**).

### Genetic diversity of *Blastocystis* subtypes

A total of 11 representative sequences, including five sequences that had been described previously and six novel sequences comprising ST10 (n = 2), ST5 (n = 1), and ST4 (n = 3), were obtained from the six *Blastocystis* subtypes. The two sequences of ST1 (Genbank number: ON834472) were identical and showed 100% similarity to a *Blastocystis* sequence isolated from humans in Turkey. ST2 (Genbank number: ON834473) and ST14 (Genbank number: ON834474) sequences were same to *Blastocystis* sequences isolated from humans in Turkey and the white-tailed deer from the USA, respectively. In the same line, 10 of the 15 sequences of ST4 (Genbank number: ON834470) were identical and showed 100% similarity to a *Blastocystis* sequence from coypu in China, one (Genbank number: ON834471) showed 100% similarity to a sequence from the Water Deer in Korea, while the remaining three had not been previously described. The ST4-1 (Genbank number: ON834467), ST4-2 (Genbank number: ON834468), and ST4-3 (Genbank number: ON834469) sequences had one base, four base, and three base differences compared to sequences isolated from humans in Thailand (Genbank number: MT947111), humans in the Netherlands (Genbank number: KF242015), and a Water Deer in Korea (Genbank number: MT114835), respectively.

The two ST10 sequences had 89.2% similarity with 57 base variations. The ST10-1 (Genbank number: ON834464) sequence had the highest similarity with that from *Cervus nippon* in Malaysia (Genbank number: KU981014) with 10 base differences, while the ST10-2 (Genbank number: ON834465) sequence had the highest similarity with that from *Cervus timorensis* in Malaysia (Genbank number: KU981005) with 12 base differences. The ST5 (Genbank number: ON834466) sequence had the highest similarity with that of pigs in the USA, with three base differences.

**Figure 1:**
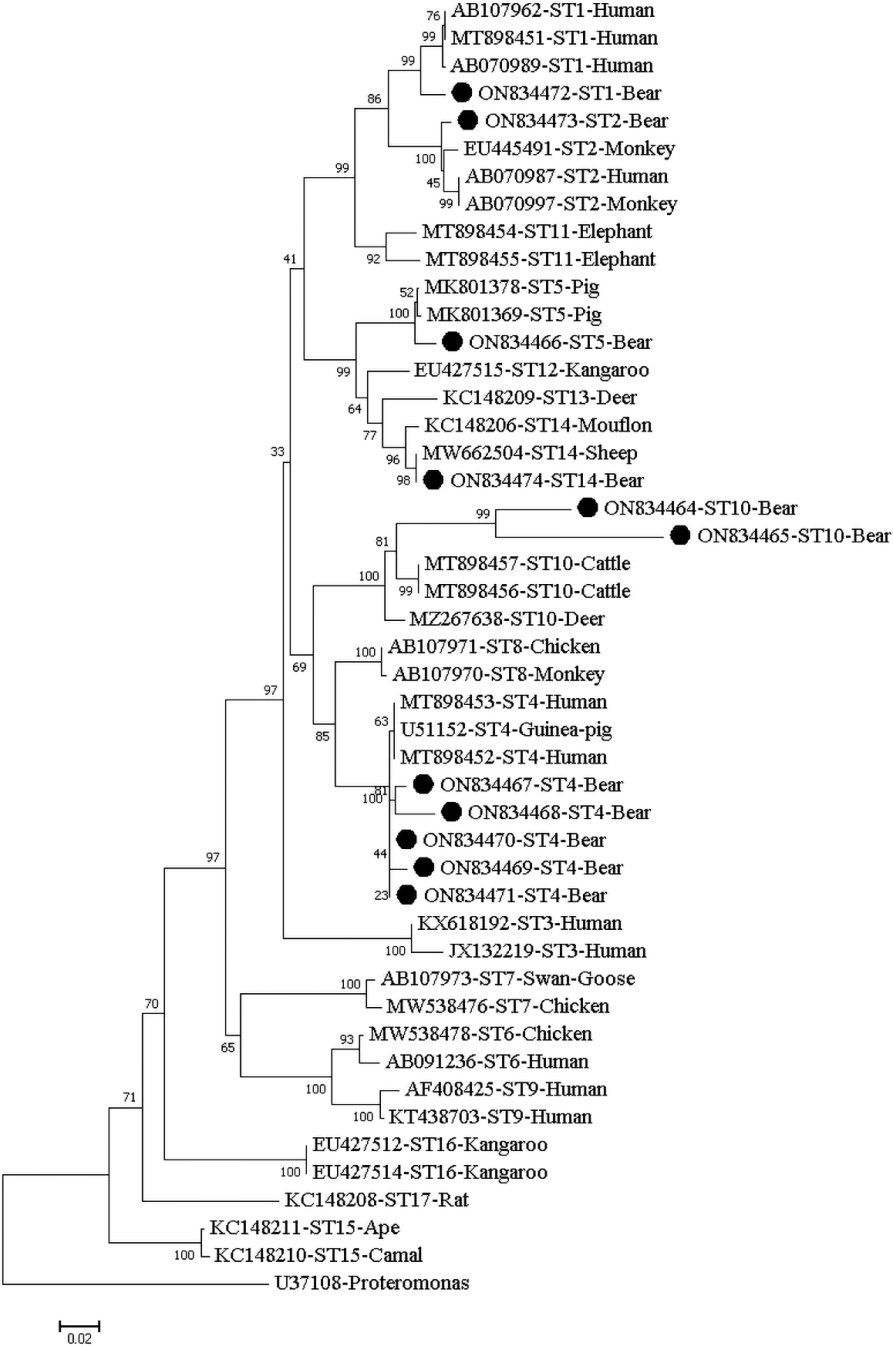
Phylogenetic relationship of the STs of *Blastocystis* isolates from fecal samples of captive Asiatic black bears. The relationship of the *Blastocystis* subtypes identified here to some known subtypes stored in the Genbank was inferred by a neighbor-joining (NJ) method based on evolutionary distances calculated using the Kimura two-parameter model. Bootstrap values were obtained using 1000 replicates. Each sequence is identified by its GenBank accession number, host origin, and ST subtype. The sequence of the ST subtype identified in the present study is indicated by a black triangle.

Isolates of the same ST clustered together in the phylogenetic tree, with ST10 isolates obtained herein clustering into a distinct evolutionary branch (**Figure 1**).

## Discussion

In the present study, 11.0% (24/218) of the animals, 9.9% on the farm and 16.7% in the zoo, were confirmed to be infected with *Blastocystis*. To date, only two studies have reported bear infection with *Blastocystis* in China (Ni et al., 2021; Deng et al., 2021). Ni et al. reported that the overall *Blastocystis* prevalence was 14.4% in farmed black bears from Heilongjiang (Ni et al., 2020). In the same line, Deng et al. reported that 25% of black bears in the zoo from Sichuan were positive for *Blastocystis* sp (Deng et al., 2021). The low infection rate of *Blastocystis* in bears in Heilongjiang province may be related to climate factors, especially rainfall. The infection rates in a specific host are related to many factors, including the immune status of the animals, examination methods, sample size, and seasonality, among other factors. We are thus unable to provide a satisfactory explanation for the actual discrepancies in the occurrence of *Blastocystis* sp. between different studies.

Previous surveys report that *Blastocystis* has a higher infection rate in young animals than in adult animals (Ni et al., 2021; Popruk et al., 2021). In the present study, both the farmed and zoo young bears had higher infection rates (23.5% and 33.3%) of *Blastocystis* than the adult bears (2.3% and 8.3%). Another study reported similar results in which young black bears (18.3%) had a significantly higher prevalence of *Blastocystis* than adult black bears (9.1%) (Ni et al., 2021). These data suggest that age may be a risk factor in bears. Besides age, feeding methods also affect the infection rate of *Blastocystis*. A previous study found that the prevalence of *Blastocystis* was significantly higher in outdoor-reared bears (24.6%) than in indoor-reared bears (12.2%) (Ni et al., 2021). Herein, the prevalence of *Blastocystis* in group feeding was 13.3%, double that of individual feeding (6.5%). This phenomenon is attributed to crowding during group feeding, which causes the animals to have more contact opportunities. The occurrence of an infection in one animal thus easily spreads to the other individuals, highlighting the importance of good hygiene in controlling *Blastocystis* infection.

In the present study, six STs (ST1, ST2, ST4, ST5, ST10, and ST14) were detected in the sampled confined Asiatic black bears. Notably, only ST1 and ST17 have been previously identified in bears (Ni et al., 2021). Herein, despite ST1 being only detected in two animals, the two came from different regions, i.e., Fujian and Heilongjiang. ST1 is the second most common ST infecting humans and has been detected in animals worldwide (Hublin et al., 2021; Popruk et al., 2021). Similarly, ST2 is the third most common ST infecting humans and is also characterized by a wide host range and a high incidence in animals (Hublin et al., 2021; Popruk et al., 2021). Detecting ST2 in bears expanded the host range of ST2. However, the transmission dynamics of ST2 and the burden of human blastocystisasis caused by ST2 and attributable to bears need to be assessed through systematic molecular epidemiological investigations of humans and animals in the future. ST4 was the most prevalent *Blastocystis* ST in this study (62.5%) and has previously been detected in humans and various animals, such as cattle, sheep, goats, deer, rodents, birds, and some wild mammals worldwide, indicating that it has a wide host range (Wang et al., 2018; Popruk et al., 2021; Hublin et al., 2021). These findings collectively suggest that bears infected with ST1, ST2, and ST4 threaten humans and other susceptible animal hosts.

The non-common STs (ST5, ST10, and ST14) have diverse geographical distributions. ST5 is probably the most frequently circulating STs among pigs, and has a 1.64% global carriage rate in human samples (Asghari et al., 2021; Hublin et al., 2021). In China, ST5 is the dominant subtype infecting artiodactyls (Ning et al., 2020). Animal-to-human transmission of ST5 has been reported in pigs and their caretakers in China and Australia (Yan et al., 2007; Wang et al., 2014). ST10 and ST14 are commonly found in livestock compared to humans (Shams et al., 2021; Hublin et al., 2021). ST10 and ST14 have been rarely reported in humans in healthy school children from Senegal, thus highlighting their low zoonotic potential (Khaled et al., 2020). Both ST10 and ST14 were detected in zoo bears herein, with the former being most predominant. It is necessitates further studies to understand the potential interspecies transmission of those two *Blastocystis* STs between bears and human as well as other animals.

In the phylogenetic tree, the same ST clustered together. Subsequently, they were grouped with their reference STs derived from humans and/or animals from different countries and the two sequences of ST10 obtained here were clustered into a distinct evolutionary branch (**Figure 1**).

Collectively, this report describes the occurrence of *Blastocystis* in farmed black bears and those from a zoo in two provinces of China, analyzes the characteristics of the *Blastocystis* STs. *Blastocystis* sp. infection with multiple zoonotic *Blastocystis* subtypes, including ST1, ST2, ST4, ST5, ST10, and ST14 in bears, is common in Heilongjiang (16.7%) and Fujian (9.9%) provinces. One health approaches need to be implemented to control and prevent the potential zoonotic transmissions.

## Data availability statement

The data presented in the study are deposited in the Genbank repository, accession number ON834464 to ON834474.

## Ethics statement

The protocol of this study was reviewed and approved by the Research Ethics Committee and the Animal Ethical Committee of Wenzhou Medical University. All fecal samples were obtained from feces defecated by animals with the approval of the animals’ owners or managers. No animals were hurt during the procedure.

## Author contributions

WZ conceived and designed the study. Y-LZ, NZ,YY and JC performed the experiments. XP, XZ, and YL analyzed the data. WZ wrote the manuscript with contributions from other authors. All authors contributed to the article and approved the submitted version.

## Funding

This work was supported by the Funded Project of Zhejiang Province University student science and Technology Innovation Activity Program (No. 2022R413A002).

## Conflict of interest

The authors declare that the research was conducted in the absence of any commercial or financial relationships that could be construed as a potential conflict of interest.

## Publisher’s note

All claims expressed in this article are solely those of the authors and do not necessarily represent those of their affiliated organizations, or those of the publisher, the editors and the reviewers. Any product that may be evaluated in this article, or claim that may be made by its manufacturer, is not guaranteed or endorsed by the publisher.
